# Clinical Practice for Venous Thromboembolism Prophylaxis in Patients Undergoing Oncological Surgeries

**DOI:** 10.7759/cureus.16627

**Published:** 2021-07-25

**Authors:** Ahmed Bilal Akhtar, Syed Raza Mehdi, Ahsun Khan, Muhammad Toqeer Zahid, Muhammad Abu Bakar

**Affiliations:** 1 Anesthesia and Critical Care, Shaukat Khanum Memorial Cancer Hospital and Research Centre, Lahore, PAK; 2 Surgery, Shaukat Khanum Memorial Cancer Hospital and Research Centre, Lahore, PAK; 3 Biostatistics and Epidemiology, Shaukat Khanum Memorial Cancer Hospital and Research Centre, Lahore, PAK

**Keywords:** venous thromboembolism, oncology, surgery, mechanical prophylaxis, pharmacological prophylaxis, audit

## Abstract

Objective

To determine the proportion of patients receiving venous thromboembolism (VTE) prophylaxis after oncological surgeries as per the hospital standards and its comparison with the international guidelines.

Methodology

In the month of September 2019, all patients after elective oncological surgeries were reviewed for VTE prophylaxis administration and education. Results were shared with the department of surgery and Hospital Quality and Patient Safety Department. Education was provided to the relevant staff and hospital policy for VTE prophylaxis was revised followed by a loop audit which was done in October 2020. The primary endpoint was to compare the proportion of patients receiving prophylaxis as per the hospital guidelines.

Results

Total 425 patients were included in this audit (209 in September 2019 and 216 in October 2020). Compliance with mechanical prophylaxis increased from 84.7 % to 98.6% and pharmacological prophylaxis improved from 39.7% (n=83) to 73.1% (n=158). Adherence to local protocols enhanced significantly from 1.9% (n=4) to 56.4% (n=122). The main cause of non-compliance was lack of risk assessment for VTE.

Conclusion

VTE prophylaxis can be improved by setting protocols in accordance with the international guidelines and local protocols. This can prevent significant morbidity and mortality in surgical patients as well as hospital costs.

## Introduction

The term venous thromboembolism (VTE) comprises of both deep vein thrombosis (DVT) and pulmonary embolism (PE) [1]. Hospital-acquired VTE covers all cases that occur in hospital and within 90 days after a hospital admission [2]. Abnormal blood clotting in deep veins of body is called deep vein thrombosis whereas pulmonary embolism occurs when the clot breaks free and blocks the arteries of lung [3]. Three main factors that may cause a venous thrombus to be formed are hypercoagulability, stasis, and endothelial injury, also known as Virchow’s Triad [4]. Moreover, some people may be more prone to develop either PE or DVT due to genetic predisposition [5,6].

The annual incidence of VTE in European origin approximately ranges between 104 to 183 per 100,000 persons [7-11]. Furthermore, the estimated annual occurrence of VTE in the United States is 1 to 2 per 1000 of the population, or 300,000-600,000 cases [11-13]. As per the Center for Disease Control and Prevention, an estimated 60,000-100,000 Americans die from VTE annually [3]. It is associated with an annual death of 25000 people and about 10% of in-hospital mortality in National Health Service (NHS) [2]. About one-third of the patients who had VTE can suffer from recurrence within 10 years. The occurrence of VTE in young population is about 1 per 100,000 and increases to 1 per 100 in ages >80 years. Men have slightly increased overall incidence of VTE than women [3].

Surgery is associated with a substantial risk of VTE, especially within the first post-operative week in comparison to the background incidence of VTE in the general population [14,15]. Although cancer is a hypercoagulable state but the risk of VTE is particularly increased in patients who are undergoing surgery (three- to fivefold) [16], who are receiving chemotherapy (6.5-fold) [17], who carry certain genetic mutations [18], and those with previous history of DVT [19]. VTE risk is especially high among certain cancer subgroups, hospitalized patients, those undergoing active antineoplastic therapy, and those receiving certain supportive care measures [20].

As per the National Institute for Health and Care Excellence (NICE) UK guidelines, all the patients should be assessed at the time of admission for risk of VTE and bleeding. A National VTE Risk Assessment Tool has been used in the NHS since last eight years to identify a person's risk for VTE. It is filled within the first 24 hours of admission. Pharmacological VTE prophylaxis for surgical and trauma patients should be started as soon as possible and within 14 hours of admission, unless otherwise stated in the population-specific recommendations [2].

Two types of prophylaxis, pharmacological and mechanical can be given for prevention of VTE. Mechanical includes Intermittent Pneumatic Compressions (IPC) and graduated compression stockings. Two metanalyses have shown that mechanical prophylaxis (especially IPC) when compared with no prophylaxis can reduce DVT (including asymptomatic) by 60% [21,22]. Most commonly used medication for VTE prevention include unfractionated heparin, low molecular weight heparin (LMWH) and fondaparinux [23]. Multiple studies have shown that DVT incidence can reduce up to 60% when mechanical prophylaxis is given in combination with pharmacological prophylaxis [24,25].

The aim of this complete audit cycle was to assess the proportion of surgical oncology patients receiving VTE prophylaxis at a tertiary care cancer hospital in accordance with established standards of practice outlined by the hospital and the international guidelines [23].

## Materials and methods

Setting

This audit was conducted at the surgical and anesthesia department of Shaukat Khanum Memorial Cancer Hospital and Research Center (SKMCH & RC) after approval from the Institutional Review Board (IRB) of hospital.

Design

It was a classical audit cycle design. First audit was done to measure the current practice of VTE prophylaxis and the compliance with hospital standards and international guidelines. The results were shared with the Hospital Quality Improvement department and methods of improving the compliance were formulated. Post-intervention loop audit was done after one year to look for improvement and adherence to the VTE prophylaxis.

Intervention

The results were shared with the surgery as well as the nursing department. The hospital policy was revised as per the new international guidelines. A pathway was established by the hospital Quality improvement department in which the patients admitted for surgery would be assessed for the risk of developing VTE. A time of 12 months was given for the above to be implemented.

Duration and sample size

Both audit cycles were done over a period of one month each. 209 patients were enrolled in the first audit cycle from 1st-30th September 2019 and 216 patients were followed in the second audit cycle from 1st-31st October 2020.

Inclusion and exclusion criteria

All the patients admitted for gastrointestinal, thoracic, gynecological, orthopedic, neurological, breast, urological, hepatobiliary, ophthalmological, head and neck oncological surgeries during the audit duration were included. Patients with age less than 18 years, day case surgery and patients with bleeding disorders were not included. There was no upper limit for age.

Hospital Policy and current practice

According to the hospital policy, all the patients planned for elective oncological surgeries had to be started on pharmacological prophylaxis pre-operatively as long as they meet the following criteria of having platelet count > 20,000, INR (if checked) < 2.0 and no evidence of active bleeding. The prophylaxis would continue up to seven days after discharge. Choice of prophylaxis may include any of the two, unfractionated heparin 5000 IU every eight hours or LMWH 40mg daily. Mechanical prophylaxis will be given peri-operatively till the patient is fully mobilized or discharged for home.

Data

The data were recorded on a predesigned proforma. Most of the information like demographics, procedure, pharmacological and mechanical prophylaxis duration and risk assessment was obtained from the Hospital Information System (HIS). Only questions about patient education were asked directly from patient.

Statistics

The data were analyzed in relation to whether the VTE risk assessment was done, prophylaxis pharmacological or mechanical was given and the duration of prophylaxis. If patient education was provided and the prophylaxis given was in accordance with the hospital policy. Statistical analysis was done using SPSS version 21.0 (IBM Corp., Armonk, NY). For continuous variables mean and median were calculated. Whereas for categorical variables frequency and percentages were calculated. All the data were categorized according to surgical subspecialties. Surgery specialties with a smaller number of cases were categorized in the “Others” group.

## Results

First audit cycle

**Figure 1:**
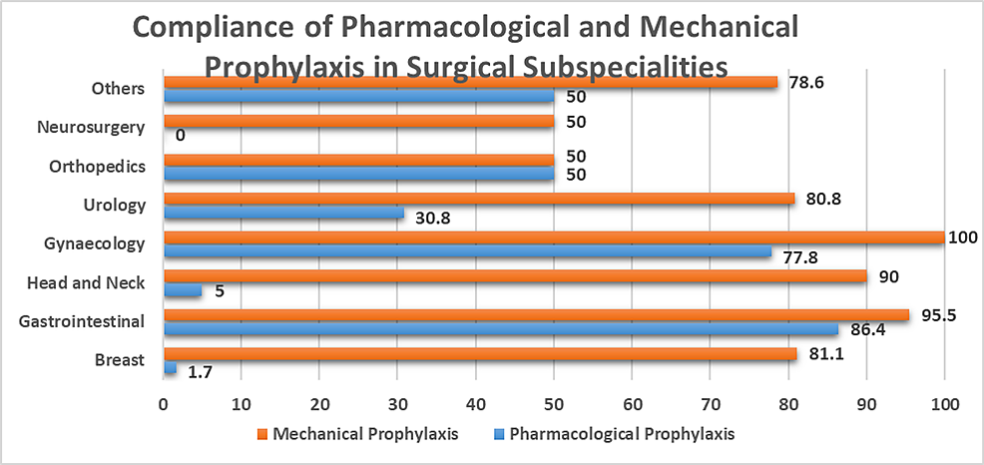
Surgery-wise comparison of both prophylaxis in audit cycle 1.

Total 209 patients were looked into the first cycle of the audit. Out of which the highest number of patients, 66 underwent gastrointestinal surgeries and only 6 patients had undergone neurosurgery. The mean age and BMI of the patients were 48.1±14.0 and 26.0±6.4. 54.5% (n=114) patients were females and 45.5% (n=95) patients were males. Figure 1 shows the compliance of both prophylaxis in surgical specialties. Pharmacological prophylaxis was given in 39.7% (n=83) patients and mechanical prophylaxis was given in 84.7% (n=177) patients. None of the patients had a risk assessment documented in HIS. Although the number of patients receiving prophylaxis was high, overall only 1.91% (n=4) of patients were given prophylaxis, both mechanical and pharmacological, as per the hospital standards. Most of the patients did not receive both the prophylaxis for the optimum time duration as mentioned above in hospital policy. There was only one case of DVT documented during that time duration. The patient did not receive pharmacological prophylaxis for VTE as recommended in the hospital policy. 

Second audit cycle

**Figure 2:**
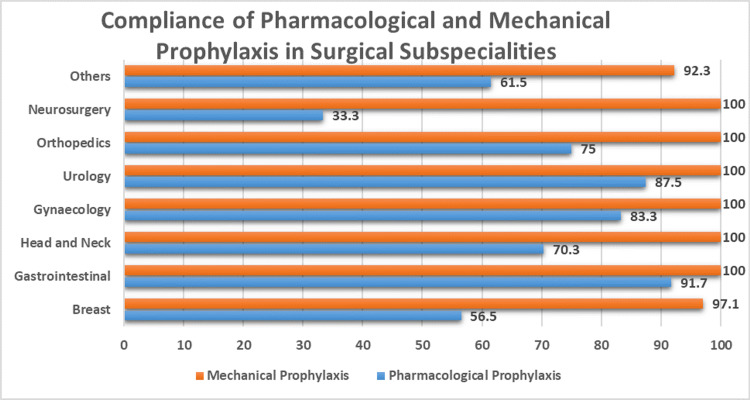
Surgery-wise comparison of both prophylaxis in audit cycle 2.

Total 216 patients were enrolled in the second cycle of the audit. There was a significant improvement in the compliance of pharmacological and mechanical prophylaxis which increased to 73.1% (n=158) and 98.1% (n=212) respectively. Adherence to hospital standards raised remarkably from 1.91% (n=4) to 56.4% (n=122) and the risk assessment was documented for 65.7% (n=142). The highest number of patients were from Breast surgery. Figure 2 shows the adherence of both prophylaxis in surgical specialties after intervention in the second audit cycle. The mean age and BMI of the patients were 45.3±13.6 and 25.8±5.6, similar to the first cycle of audit. 62% (n=134) patients were females and 45.5% (n=95) patients were males. No case of VTE was documented in that time period.

Table 1 shows the comparison of patient data and compliance to prophylaxis between both the audit cycles. The reasons identified for not following hospital protocols are mentioned in Table 2 with the respective percentages.

**Table 1:** Comparison of audit cycles 1 and 2.

Parameters	Audit cycle 1 (n=209)	Audit cycle 2 (n=216)
Age (mean ± SD)	48.1±14.0	45.3±13.6
Males (n, %)	95 (45.5%)	95 (45.5%)
BMI (mean ± SD)	26.0±6.4	25.8±5.6
Mechanical prophylaxis (n, %)	177 (84.7%)	212 (98.1%)
Pharmacological prophylaxis (n, %)	83 (39.7%)	158 (73.1%)
Compliance to hospital policy (n, %)	4 (1.91%)	122 (56.4%)
Risk assessment documentation (n, %)	0	142 (65.7%)

**Table 2:** Reasons for non-compliance.

Reasons of non-compliance	Pharmacological		Mechanical	
	First audit (total 209)	Second audit (total 216)	First audit (total 209)	Second audit (total 216)
Not given (n, %)	126 (60.3%)	58 (26.9%)	32 (15.3%)	4 (1.9%)
Late initiation (n, %)	32 (15.3%)	11 (5.1%)	4 (1.9%)	0
Early discontinuation (n, %)	50 (23.9%)	18 (8.3%)	2 (0.9%)	0
Inappropriate dose (n, %)	6 (2.9%)	2 (0.9%)	-	-
Inappropriate drug/method (n, %)	5 (2.4%)	0	0	0

## Discussion

In this complete audit cycle, we identified the lapses in the current practice of VTE risk assessment and compliance, subsequent feedback was given and methods for improving the outcome were implemented. Although most of the surgical patients were receiving VTE prophylaxis but on detailed account of individual assessment of patients record revealed that about 98% were not receiving prophylaxis in accordance to the hospital policy and international guidelines. 15.3% of the patients did not receive any prophylaxis at all and out of those who had been given prophylaxis, only 39.7% received both the type of prophylaxis. In addition to that, the duration of prophylaxis given was not as per the hospital standards causing the percentage adherence for the policy to be minimal.

The main reason for low compliance to thromboprophylaxis at our hospital was lack of VTE risk assessment before surgery in audit cycle one. The risk assessment was still 65.7% in audit cycle two warranting improvement. Other causes for inadequacy of prophylaxis included late beginning of therapy, in appropriate dose, no prophylaxis at all and in appropriate drugs. If we compare our results with other studies, Yu et al. [26] reported a compliance rate of 13.3% and omission of prophylaxis as the main reason for not following the guidelines. As per the audit of Yu et al, only 12.7% of patients of General Surgery group and 9.9% of urology group received prophylaxis according to the American College of Chest Physicians guidelines. However, the numbers were even less for urology and Gastrointestinal surgeries in our audit.

Many studies have shown that the application of simple methods like regular auditing on VTE prophylaxis and practitioner education can change the outcome completely [27,28]. Following methods have been used for improving the adherence to VTE prophylaxis such as monthly or bi-monthly meetings between the hospital’s surgical and anesthesia executives, risk assessment prompts on Hospital Information System, posters in wards where surgical patients are admitted and production of laminated cards or handouts incorporating the hospital’s thromboprophylaxis guidelines [27,28].

Our audit had several limitations such as small sample size, lack of comparison and randomization (sample was continuous), single centered and the design of the study did not permit significant statistical analysis. In addition to that, the continuous change in staff including doctors and nurses dealing with patients’ peri-operatively did not permit a controlled environment to assess the results of teaching and feedback system. Although the compliance improved after intervention, the results could have been more significant. Furthermore, the lack of assessing all patients for silent VTE could have given an insight into the unidentified cases and reinforced the importance of prophylaxis.

Also, we did not consider the comorbid conditions patients were having and the nature of surgery which may have restricted the use of pharmacological prophylaxis. However, we did exclude patients already diagnosed with a bleeding disorder but it did not completely rule out bleeding due to comorbid conditions. This is another reason that a proper VTE risk assessment should be documented for every patient regardless of the cause of admission to the hospital. By taking simple measures as such, significant morbidity and mortality can be decreased.

## Conclusions

In conclusion, at SKMCH & RC compliance of thromboprophylaxis was low in the first audit cycle but after intervention the results improved significantly. However, there is room for more improvement and such audits should be conducted on regular bases to evaluate and elevate the standards of practice in the hospital.
